# Changes in proprioception at different time points following anterior cruciate ligament injury or reconstruction

**DOI:** 10.1186/s13018-023-04044-5

**Published:** 2023-07-31

**Authors:** Yixuan Zhao, Ze Chen, Longfei Li, Xipeng Wu, Wei Li

**Affiliations:** 1 School of Rehabilitation Medicine, Binzhou Medical University, Yantai, Shandong China; 2 Tianjin Medical University, Tianjin, China; 3 Department of Rehabilitation, Binzhou Medical University Hospital, Binzhou, Shandong China

**Keywords:** ACL, ACLR, Proprioception, Rehabilitation, Repair

## Abstract

**Purpose:**

To investigate the changes in 30° and 60° position sense in patients with anterior cruciate ligament (ACL) injury at different time points after injury and reconstruction.

**Methods:**

Patients were divided into six groups according to time after ACL injury and reconstruction: group A (ACL injury 1.5–6 months), group B (ACL injury 6–12 months), group C (ACL injury > 12 months), group D (postoperative ACL reconstruction 1–6 months), group E (postoperative ACL reconstruction > 6 months), and group F consisting of 14 healthy adults (control group). The ability of the affected leg to reproduce the same joint position during knee flexion was tested using active joint position sense assays to assess proprioception in both the lower extremities of the patient or between groups.

**Results:**

Proprioception decreased rapidly during the early stages of ACL injury. Significant difference in the affected side at 30° compared to the healthy side (Group A: 4.70 (4.78, 9.00) vs 4.15 (3.35, 6.13), *P* = 0.03; Group B: 2.90 (0.48, 4.56) vs 8.30 (4.18, 10.43), *P* = 0.001; Group E: 6.25 (2.55, 11.60) vs 9.60 (3.90, 12.73), *P* = 0.009). However, no significant differences were detected for a double lower limb contrast of 60° (Group A: 5.1 (1.00, 8.00) vs 3.00 (0.75, 3.55), *P* = 0.044). Finally, the affected side of patients in groups C, D and E had significant differences in position perception at 30° compared with healthy subjects (*P* < 0.01), and the affected side of patients in groups C and E had significant differences in position sense at 60° compared with healthy subjects (*P* < 0.01).

**Conclusion:**

ACL injury had a greater impact on the patient's 30° position sense, with only a small impact for 60°. Further, the early and middle proprioception recovery stages after ACL injury were the best before surgery. Finally, proprioception recovery training should be performed soon after injury.

## Introduction

The anterior cruciate ligament (ACL) plays a major role in maintaining knee-joint stability by contributing to both the functionality and mechanical congruence of the lateral and medial tibiofemoral joints [1]. ACL injury is one of the most common sports injuries [2, 3]. Currently, most researchers believe that anterior cruciate ligament reconstruction (ACLR) is the best treatment method for ACL injuries. However, a rate of secondary ACL injuries of up to 30% has been reported in the 15 years after reconstruction [4]. This may be related to ACL proprioception. As ACL injury damages the stability of the knee joint, loss of proprioceptive function affects the range of motion and stability of the joint, leading to injury of the articular cartilage, meniscus, and other important structures, as well as secondary traumatic osteoarthritis, seriously affecting the function of the knee joint [5].

In addition to its function as a knee stabilizer, the ACL also has proprioceptive functions [6, 7]. Proprioception, the sensory modality responsible for the sensation of joint motion and position, plays a crucial role in the control of arc and normal joint performance by the afferent–efferent neuromuscular system [8]. Proprioceptive receptors in the muscles, skin, and joints convey afferent sensorial information to the brain, including tension, force, force movement, and position [9]. The ACL is an important structure responsible for knee proprioception [10]. Papalia et al. [11] believed that ACL injuries may lead to alterations in knee stability, not only due to biomechanical changes caused by ligament deficiency, but also due to proprioceptive changes caused by the knee structure. As such, an intact ACL is critical for providing both static and dynamic stability to the knee, as well as proprioceptive regulation of the knee joint [12].

ACL injury leads to knee dysfunction, primarily because the number of proprioceptors on the ACL decreases, tissue destruction around the knee joint causes abnormal proprioceptive afferents, and the knee joint cannot perceive joint location and transmit motor sensation signals [13]. As ligament injuries are difficult to heal, most patients choose ACLR after ACL injury to restore knee biomechanical stability [14]. However, patients still have deficits in proprioception and often experience symptoms such as knee instability, slow reaction time, dynamic balance control, and decreased coordination after ACL reconstruction [15]. This can lead to a loss of motor control acuity such that there will be a decline in overall stability of the knee joint, joint movement coordination, balance, and flexibility. It is widely accepted that ACL injury impairs neuromuscular control and proprioceptive acuity of the knee joint [16]. As such, proprioceptive acuity testing of the knee joints is common.

Hohmann et al. [17] believed that knee flexion motor sensation is provided by mechanoreceptors in ligament around the knee joint, the ACL is considered to be the primary driver with knee flexion function. As such, it is reasonable to infer that knee flexion can examine the ACL proprioceptive situation. Among the proprioceptive senses, joint position sense (JPS) is one of the most commonly tested, typically involving passive or active reproduction of joint angles with a confounding sense of vision occluded [18].Thus, in the present study, we performed bilateral lower limb active knee 30° and 60° position perception testing by synchronously recording patients with ACL injuries [19–21]. We further analyzed the differences in proprioception after ACL injury between the affected and healthy sides and clarified the characteristics of the proprioceptive changes caused by ACL injury. The purpose of this study was to determine whether proprioception decreases after unilateral ACL injury by exploring the degree of sensory changes at the 30° and 60° positions, to thereby provide a theoretical basis for treatment of ACL injuries, inadequate knee stability, and rehabilitation.

## Methods

### General information

Eighty-four adults with unilateral ACL injuries, ranging in age from 18 to 45 years, were recruited for the study between October 1, 2021, and August 1, 2022. Patients were divided into five groups based on the time since injury: group A (preoperative ACL reconstruction, injury 1.5–6 months), group B (preoperative ACL reconstruction, injury 6–12 months), group C (preoperative ACL reconstruction, injury > 12 months), group D (postoperative ACL 1–6 months), and group E (postoperative ACL > 6 months). Group F consisted of 14 healthy adults (average age: 23 years) and was regarded as the control group (Table 1).

**Table 1:** Classification of characteristics

Classification	Group A	Group B	Group C	Group D	Group E	Group F
N	14	14	14	14	14	14
Gender (F/M)	5/9	9/5	10/4	11/3	10/4	6/8
Age (years)	25.25 ± 11.62	28.0 ± 2.80	32.33 ± 5.66	28.20 ± 7.25	29.35 ± 7.52	22.62 ± 1.29
Weight (kg)	63.63 ± 7.83	68.58 ± 11.87	75.25 ± 12.13	73.03 ± 11.80	78.78 ± 14.83	61.77 ± 9.11
Height (cm)	165.88 ± 9.03	172.83 ± 7.32	172.33 ± 9.10	173.07 ± 7.31	174.95 ± 9.32	168.0 ± 9.78
BMI (kg/m^2^)	23.12 ± 1.80	23.0 ± 2.55	25.17 ± 1.82	24.34 ± 2.81	25.65 ± 3.07	21.76 ± 2.72
Affected side (L/R)	10/4	7/7	9/5	10/4	6/8	10/4

### Inclusion and exclusion criteria

The inclusion criteria were as follows: (1) MRI showing simple ACL injury with good ligament tissue structure; (2) no meniscal injury; (3) no presence of internal tumors, infection, fracture; (4) no mental illness; (5) no unstable vital signs in major organs such as the heart, brain, and kidney; (6) no ACL injury secondary to immune and metabolic diseases; (7) no severe osteoporosis; (8) no venous thrombosis; (9) not pregnant or lactating women; (10) informed consent and voluntary cooperation of patients; (11) unilateral knee injury.

The exclusion criteria were as follows: (1) Limited joint movement prevents accurate testing; (2) patients unable to complete evaluation and rehabilitation training as required.

### Knee joint position perception measurement

**Fig. 1:**
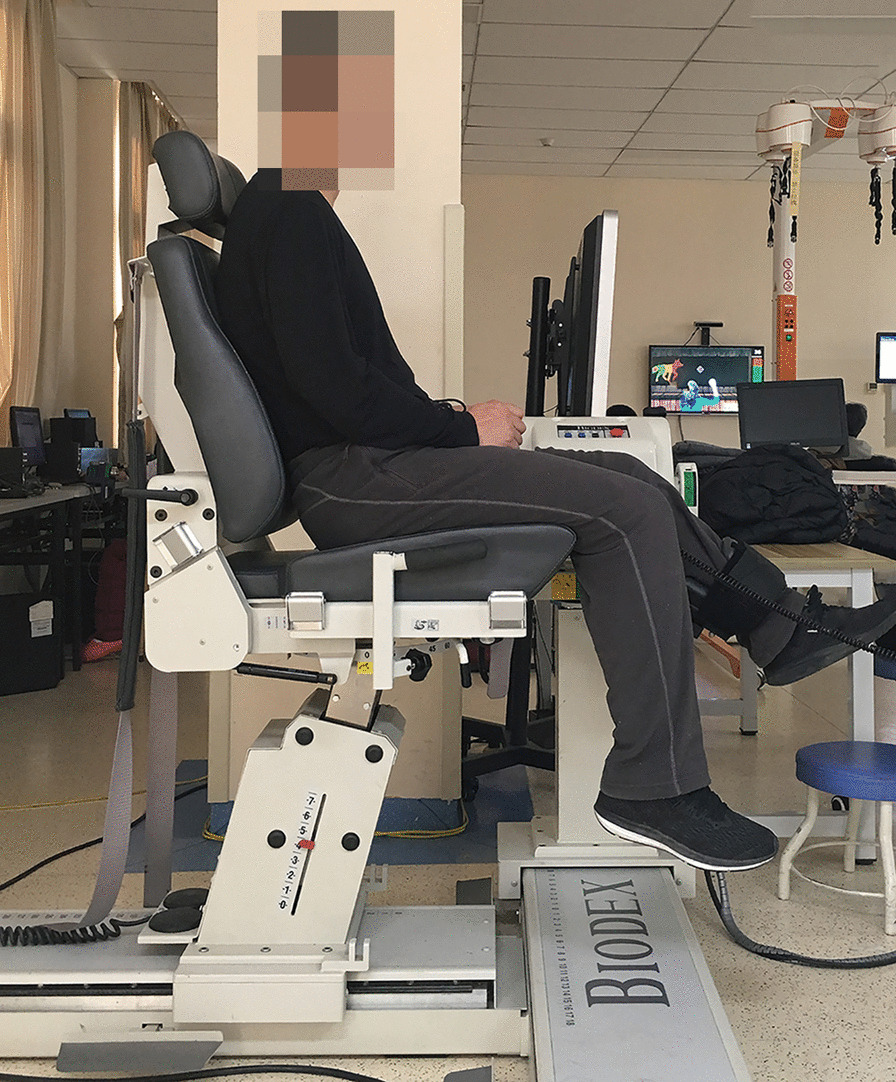
Biodex medical system

Joint angle error testing is a viable method to assess clinical joint proprioception [22], which can be used to accurately determine the position of a specific body part in space by measuring the degree of angular deviation from the starting position [23]. Our study assessed the proprioceptive state using the Biodex Medical System 4 (Biodex Medical System, New York, NY, USA) with eyes closed for JPS using the Active Angle Reproduction (AAR) technique [24] (Fig. 1). The outcome variables were measured in the following four trials: (1) measurement of affected side 30°; (2) measurement of unaffected side 30°; (3) measurement of affected side 60°; and (4) measurement of unaffected side 60°. We have been trained as orthopedic rehabilitation therapists prior to the start of the trial, and after achieving a uniform standard, two senior physicians from the rehabilitation assessment team conducted a position perception assessment.

The specific operations were as follows: The knee was moved from a 90° flexion starting position passively to each of the target angles of 30° and 60°. We have reminded patients to close their eyes before collecting data. Please patient hold the leg in the 30° knee extension and 60° knee extension positions for 10s to allow the patient to memorize the position, and was then returned to 90° knee flexion. After a pause of 10 s, the patient, with the memory of the active knee flexion, moves the lower limb in the same way by active contractions and stops when the patient perceives that the target angle has been reached. The mean values of the six trials were obtained for each patient at each angle and used to calculate the difference between the actual angle achieved and the target angle [25]. The smaller the difference, the better the patient's perception of the position [26].

### Statistical methods

The sample size was estimated using the G-power 3.1 (Heinrich Heine University Düsseldorf, Germany) calculator. Considering a 95% significance level and 80% power, we calculated the sample size to be 78. However, taking into account the potential dropouts of some patients or unavailability of data, a total of 90 samples we were tested and 84 samples were finally selected. Data were processed using SPSS 22.0. Comparisons between the patients’ lower limbs were performed using the paired t test for normally distributed quantitative variables, while the Wilcoxon signed-rank sum test was used for non-normally distributed quantitative variables. To compare the data between groups, two independent sample t tests were used for those with normal distribution and the Wilcoxon rank sum test was used for those without normal distribution. Data plots were obtained using GraphPad Prism 8 software.

## Results

### Comparison of bilateral lower limb position perception in patients with ACL injury

The 30° position perception of lower limbs in patients with ACL injury was statistically significant (*P* < 0.05) 1.5–12 months (Group A and B) after injury, as well as 6 months (Group E) after reconstruction, Group A has poorer position perception on the affected side than on the unaffected side, while Group B and Group E have better position perception on the affected side than on the unaffected side (Table 2). However, the 60° position perception from the lower limbs of patients with ACL injury was statistically significant (*P* < 0.05) only between 1.5 and 6 months (Group A) after ACL injury, and the affected side of group A is worse than the unaffected side (Table 3).

**Table 2:** 30° position sense test results

	30° affected side	30° unaffected side	*P* value
Group A	4.70 (4.78, 9.00)	4.15 (3.35, 6.13)	0.03*
Group B	2.90 (0.48, 4.56)	8.30 (4.18, 10.43)	0.001**
Group C	9.55 (2.25, 13.78)	8.95 (3.35, 16.48)	0.46
Group D	7.70 (3.85, 12.30)	7.60 (4.10, 12.10)	0.56
Group E	6.25 (2.55, 11.60)	9.60 (3.90, 12.73)	0.009**

Statistical description: Median (interquartile difference)

*P < 0.05, significantly different, **P < 0.01, extremely significantly different

**Table 3:** 60° position sense test results

	60° affected side	60° unaffected side	*P* value
Group A	5.1 (1.00, 8.00)	3.00 (0.75, 3.55)	0.04*
Group B	4.90 (1.83, 5.90)	1.65 (1.20, 3.98)	0.05
Group C	2.10 (1.00, 4.50)	2.00 (1.38, 3.30)	0.68
Group D	3.60 (1.60, 6.15)	3.1 (1.45, 5.45)	0.30
Group E	2.55 (1.40, 4.50)	3.1 (1.23, 5.08)	0.68

Statistical description: Median (interquartile difference)

*P < 0.05, significantly different

### Comparison of position perception between groups in patients with ACL injury

#### Comparison of 30° affected side position perception between groups

**Fig. 2:**
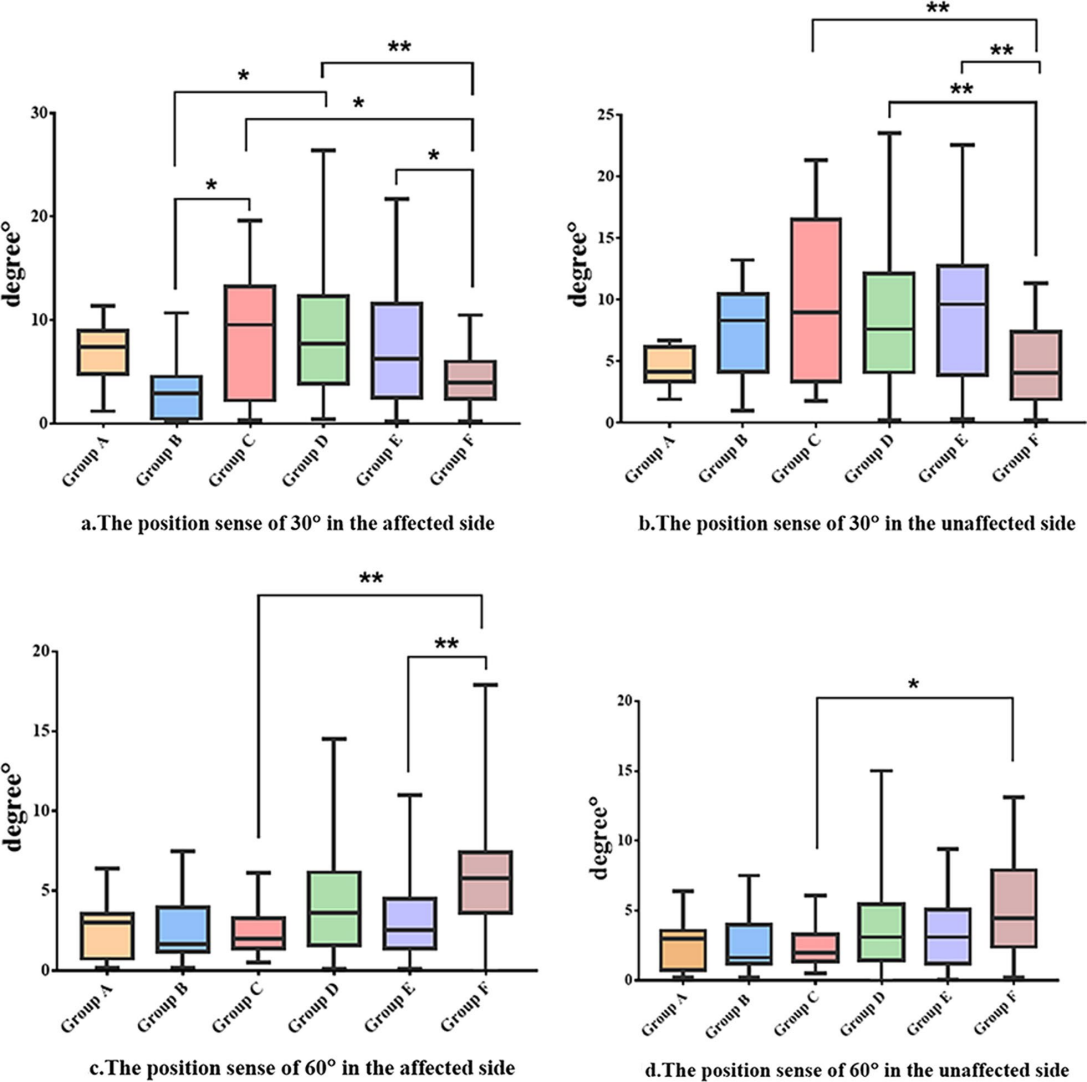
Comparison of bilateral lower limb position perception in patients with ACL injury

The comparison between 6–12 months after ACL injury and > 12 months after ACL injury was statistically significant (Group B and C) (*P* < 0.05), and Group B has a better sense of position than Group C. As was the comparison between the groups 6–12 months after ACL injury and 0–6 months after reconstruction (Group B and D), and Group B has a better sense of position than Group D (*P* < 0.05). Statistically significant (*P* < 0.05) in the ACL injury over 12 months group compared to healthy individuals (Group C and F), and Group C has a worse sense of position than Group F. Further, there was a statistically significant difference (*P* < 0.05) between the healthy group and the ACL reconstruction group more than 6 months after surgery (Group E and F), and Group E has a worse sense of position than Group F. A significant difference (*P* < 0.01) was also found between the healthy group and the ACL reconstruction group 1–6 months after surgery (Group D and F), and Group D has a worse sense of position than Group F (Fig. 2a).

#### Comparison of 30° unaffected side position perception between groups

There were statistically significant differences (*P* < 0.01) between the uninjured side and the healthy person (Group F) in the three groups with ACL injury greater than 12 months (Group C), 0–6 months after ACL reconstruction (Group D), and ACL reconstruction greater than 6 months (Group E), and the proprioception of group C, D and E was worse than that of group F (Fig. 2b).

#### Comparison of 60° affected side position perception between groups

Only ACL injury longer than 12 months (Group C) and ACLR longer than 6 months (Group E) were statistically significant (*P* < 0.01) compared with healthy person (Group F), and the results showed that groups C and E had better proprioception than group F (Fig. 2c).

#### Comparison of 60° unaffected side position perception between groups

A significant difference (*P* < 0.05) was found between healthy and uninjured patients with ACL injury greater than 12 months (Group C and F), and the results showed that groups C had better proprioception than group F (Fig. 2d).

## Discussion

We studied the changes in proprioception in patients at different times after ACL injury or ACLR. Our research found that in the 30° positional sense, the unaffected side became progressively worse over time, while positional sense on the affected side decreased between 1.5 and 6 months of injury (group A) and improved briefly between 6 and 12 months (group B) with professional rehabilitation. However, after more than one year of injury (group C), the position sense decreases again. In terms of overall trends, both the affected and unaffected side position sense are in a state of decline. Even after ACLR there was no significant improvement, and a continuous deterioration on the unaffected side. In contrast, 60° position sense only decreases at the beginning of the injury. This indicated that ACL injury has more influence on patients' sense of position at 30°, and less influence on patients' sense of position at 60°. Indeed, Zhang et al. showed that the perception of 30° position on the affected side decreased in the early stage of ACL injury, and this phenomenon exacerbated over time [27]. This may be because the ACL is stressed more when flexion is 30° with quadriceps contractions and less flexion (e.g., quadriceps isometric contractions, squats, active knee extensions). When flexion is 60°, the stress on the ACL is small as the hamstring muscle contracts and the flexion degree is large (such as the isometric contraction of the hamstring muscle, the 60° or 90° long contraction of the femoral quadriceps muscle, and the synergic contraction of the quadriceps muscle and the hamstring muscle).

Moreover, we found that the proprioception of the affected side was significantly worse than that of the unaffected side in the same individual in this study. This agrees with a study by Hu [28], which found that knee proprioception in the ACL injured knee was significantly poorer than in with contralateral unaffected knee of the same individual. Several meta-analyses have also concluded that in the same individual, the JPS error of the knee on the injured ACL side is significantly larger than that on the unaffected side [1, 29]. Although the evidence for ACL injury and JPS defects in the ACLR knee is inconclusive, existing studies have demonstrated that ACL injury can lead to decreased proprioception on the affected side. This may be because ACL injury can affect the balance and coordination of the patient's movement, resulting in decreased motor control, knee stability, coordination, balance, and flexibility, and therefore decreased proprioception.

Although several studies have demonstrated better proprioception on the healthy side than on the affected side after ACL injury or reconstruction, it has also been shown that ACL injury not only decreases proprioception on the affected side, but also affects proprioception on the healthy side. Li et al. similarly found that the unaffected side would decline with the decrease in proprioception on the affected side one year after ACL injury [30]. Other studies have shown that ACL injury not only leads to unilateral proprioception loss, but also to contralateral knee joint proprioception loss [23]. Overall, the results of these studies are consistent with our results. From the overall trend of our research and the literature, it can be said that the position perception of the undamaged side decreases with the decrease of the damaged side, both at 30° and 60°. Most importantly, decreased proprioception on both sides leads to an increased risk of secondary injury. Therefore, rehabilitation training after ACL injury or reconstruction should take into account both sides.

We found that positional awareness improved after ACLR compared to preoperative, but did not fully recover. And in the long term, the positional sense on the affected side gradually improved, while the positional sense on the unaffected side gradually deteriorated. This suggests that although ACLR is one of the main treatments for ACL injuries, proprioception is not fully restored after ACLR, which is consistent with the findings of several previous studies. Young et al. [31] argued that ACLR improved clinical motor function, but proprioception did not return to normal compared with the state before injury. Staples et al. [32] showed that although ACLR improved postural stability compared to preoperatively, proprioceptive deficits in the posterior knee of ACLR persisted for up to two years compared to the contralateral limb. The study of Nagelli et al. [16] indicated that the sensory disturbance caused by ACLR is unlikely to completely recover, and that joint efforts may be needed to compensate for the loss of ACL mechanoreceptors during rehabilitation. From the trend chart, we found that position perception on both the affected and unaffected sides deteriorated rapidly after injury, with no significant improvement after surgery. From a bilateral comparison, one year after surgery, the affected side had improved position perception compared to the unaffected side. This suggests that ACLR does play a role in improving proprioception, but does not fully restore ACL proprioception to its preoperative state. Proprioceptive training after ACLR has further been reported to help restore the static and dynamic stable structure of the knee joint [13]. Jiang et al. [33] similarly confirmed that proprioceptive training was beneficial to improve knee function and proprioception after ACLR, which suggests that proprioceptive training should be conducted as soon as possible after ACLR. Therefore, in order to improve the proprioception and knee stability of ACL patients, proprioception training should be improved in clinical practice to promote the recovery of proprioception function.

Finally, it is worth noting that in our comparison between patients with ACL and healthy subjects, we found that patients with ACL injury had a better 60° position perception than healthy subjects on the same side of the lower limb. Although previous studies have shown poor 30° position in patients with ACL injury, our current data show enhanced 60° position perception. We believe that this may have to do with the body's compensatory mechanisms. When the 30° position sensory area deteriorated, the 60° position sensory area was compensated and enhanced. But this idea has not been proven yet, and further tests are needed to confirm it.

In this study, we only studied the position perception at 30° and 60° after ACL injury, and future studies can explore the position perception changes at other angles. Moreover, our study results show that patients' 30° position sense decreases significantly, while patients' 60° position sense is better than healthy people, and the reasons for this need to be further explored in future studies.

### Study limitations

This study had several limitations which should be addressed. Firstly, we therefore need a large amount of data and long-term studies to further confirm the findings of our study. Moreover, the angle we tested was only a reference to previous studies and did not further explore other angles of proprioceptive change, which will need to be refined in subsequent studies.

## Conclusion

In conclusion, this study showed that both the proprioception of the affected and control sides decreased after ACL injury, indicating that it is difficult to return to the pre-injury state even after ACLR.

## Data Availability

Data are available on request in personal repository.

## Abbreviations

AAR: Active angle reproduction
ACL: Anterior cruciate ligament
ACLR: Anterior cruciate ligament reconstruction
JPS: Joint position sense

## References

[1] Fleming JD, Ritzmann R, Centner C (2022). Effect of an anterior cruciate ligament rupture on knee proprioception within 2 years after conservative and operative treatment: a systematic review with meta-analysis. Sports Med.

[2] Simonson R, Piussi R, Högberg J (2023). Effect of quadriceps and hamstring strength relative to body weight on risk of a second ACL injury: a cohort study of 835 patients who returned to sport after ACL reconstruction. Orthop J Sports Med.

[3] Fältström A, Hägglund M, Kvist J, Mendonça LD (2023). Risk factors for sustaining a second ACL injury after primary ACL reconstruction in female football players: a study investigating the effects of follow-up time and the statistical approach. Sports Med Open.

[4] Leys T, Salmon L, Waller A (2012). Clinical results and risk factors for reinjury 15 years after anterior cruciate ligament reconstruction: a prospective study of hamstring and patellar tendon grafts. Am J Sports Med.

[5] Zhang S, Huang Q, Xie J (2018). Factors influencing postoperative length of stay in an enhanced recovery after surgery program for primary total knee arthroplasty. J Orthop Surg Res.

[6] El-Desouky MA, Ezzat M, Abdelrazek BH (2022). Clinical outcomes in stump-preserving versus stump-sacrificing anterior cruciate ligament reconstruction; a randomized controlled study. BMC Musculoskelet Disord.

[7] Markatos K, Kaseta MK, Lallos SN (2013). The anatomy of the ACL and its importance in ACL reconstruction. Eur J Orthop Surg Traumatol.

[8] Relph N, Herrington L, Tyson S (2014). The effects of ACL injury on knee proprioception: a meta-analysis. Physiotherapy.

[9] Guney-Deniz H, Harput G, Kaya D, Nyland J, Doral MN (2020). Quadriceps tendon autograft ACL reconstructed subjects overshoot target knee extension angle during active proprioception testing. Knee Surg Sports Traumatol Arthrosc.

[10] Bartels T, Brehme K, Pyschik M (2019). (2019) Postural stability and regulation before and after anterior cruciate ligament reconstruction—a two years longitudinal study. Phys Ther Sport.

[11] Papalia R, Franceschi F, Tecame A (2015). Anterior cruciate ligament reconstruction and return to sport activity: postural control as the key to success. Int Orthop.

[12] Bartels T, Brehme K, Pyschik M (2018). Pre- and postoperative postural regulation following anterior cruciate ligament reconstruction. Exerc Rehabil.

[13] Röijezon U, Clark NC, Treleaven J (2015). Proprioception in musculoskeletal rehabilitation. Part 1: Basic science and principles of assessment and clinical interventions. Man Ther.

[14] Sanders TL, Maradit Kremers H, Bryan AJ (2016). Incidence of anterior cruciate ligament tears and reconstruction: a 21-year population-based study. Am J Sports Med.

[15] Culvenor AG, Alexander BC, Clark RA (2016). Dynamic single-leg postural control is impaired bilaterally following anterior cruciate ligament reconstruction: implications for reinjury risk. J Orthop Sports Phys Ther.

[16] Nagelli CV, Hewett TE (2017). Should return to sport be delayed until 2 years after anterior cruciate ligament reconstruction? Biological and functional considerations. Sports Med.

[17] Hohmann E, Tetsworth K, Glatt V (2019). The hamstring/quadriceps ratio is an indicator of function in ACL-deficient, but not in ACL-reconstructed knees. Arch Orthop Trauma Surg.

[18] Hillier S, Immink M, Thewlis D (2015). Assessing proprioception: a systematic review of possibilities. Neurorehabil Neural Repair.

[19] Jebreen M, Sole G, Arumugam A (2023). Test-retest reliability of a passive joint position sense test after ACL reconstruction: influence of direction, target angle, limb, and outcome measures. Orthop J Sports Med.

[20] Lee DH, Lee JH, Ahn SE, Park MJ (2015). Effect of Time after Anterior Cruciate Ligament Tears on Proprioception and Postural Stability. PLoS ONE.

[21] Alshahrani MS, Reddy RS, Tedla JS, Asiri F, Alshahrani A (2022). Association between kinesiophobia and knee pain intensity, joint position sense, and functional performance in individuals with bilateral knee osteoarthritis. Healthcare (Basel).

[22] Balke M, Liem D, Dedy N (2011). The laser-pointer assisted angle reproduction test for evaluation of proprioceptive shoulder function in patients with instability. Arch Orthop Trauma Surg.

[23] Xu J, Zhou X, Guo X (2018). Effects of unilateral electroacupuncture on bilateral proprioception in a unilateral anterior cruciate ligament injury model. Med Sci Mont.

[24] Kaya D, Guney-Deniz H, Sayaca C (2019). Effects on lower extremity neuromuscular control exercises on knee proprioception, muscle strength, and functional level in patients with ACL reconstruction. Biomed Res Int.

[25] Selfe J, Callaghan M, McHenry A (2006). An investigation into the effect of number of trials during proprioceptive testing in patients with patellofemoral pain syndrome. J Orthop Res.

[26] Suner Keklik S, Güzel N, Çobanoğlu G, Kafa N, Ataoğlu MB, Öztemür Z (2021). Evaluation of proprioception in patients who underwent ACL reconstruction: measurement in functional position. Turk J Med Sci.

[27] Zhang L, Qi J, Zeng Y (2018). Proprioceptive changes in bilateral knee joints following unilateral anterior cruciate ligament injury in cynomolgus monkeys. Med Sci Monit.

[28] Hu S, Ma X, Ma X (2023). Relationship of strength, joint kinesthesia, and plantar tactile sensation to dynamic and static postural stability among patients with anterior cruciate ligament reconstruction. Front Physiol.

[29] Strong A, Arumugam A, Tengman E (2021). Properties of knee joint position sense tests for anterior cruciate ligament injury: a systematic review and meta-analysis. Orthop J Sports Med.

[30] Li W, Li Z, Qie S (2020). Biomechanical evaluation of preoperative rehabilitation in patients of anterior cruciate ligament injury. Orthop Surg.

[31] Young SW, Valladares RD, Loi F, Dragoo JL (2016). Mechanoreceptor reinnervation of autografts versus allografts after anterior cruciate ligament reconstruction. Orthop J Sports Med.

[32] Staples JR, Schafer KA, Smith MV (2020). Decreased postural control in patients undergoing anterior cruciate ligament reconstruction compared to healthy controls. J Sport Rehabil.

[33] Ma J, Zhang D, Zhao T (2021). The effects of proprioceptive training on anterior cruciate ligament reconstruction rehabilitation: A systematic review and meta-analysis. Clin Rehabil.

